# Efficacy and Safety of Sorafenib or Lenvatinib for Advanced Hepatocellular Carcinoma after Failure of First-Line Atezolizumab Plus Bevacizumab: A Systematic Review and Meta-Analysis

**DOI:** 10.3390/cancers16162813

**Published:** 2024-08-10

**Authors:** Tzu-Rong Peng, Yi-Fang Weng, Ta-Wei Wu, Chao-Chuan Wu, Yi-Chun Chou, Ching-Sheng Hsu

**Affiliations:** 1 Department of Pharmacy, Taipei Tzu Chi Hospital, Buddhist Tzu Chi Medical Foundation, New Taipei City 231, Taiwan; tzu.rong@tzuchi.com.tw (T.-R.P.); u108534011@gap.kmu.edu.tw (Y.-F.W.); tawei@tzuchi.com.tw (T.-W.W.); 2 Department of Surgery, Taipei Tzu Chi Hospital, Buddhist Tzu Chi Medical Foundation, New Taipei City 231, Taiwan; ngchiukwan@yahoo.com.tw; 3 School of Medicine, Tzu Chi University, Hualien 97004, Taiwan; swankyblade@yahoo.com.tw; 4 Division of Gastroenterology, Department of Internal Medicine, Dalin Tzu Chi Hospital, Buddhist Tzu Chi Medical Foundation, Chia-Yi 62247, Taiwan; 5 Liver Diseases Prevention and Treatment Center, Dalin Tzu Chi Hospital, Buddhist Tzu Chi Medical Foundation, Chia-Yi 62247, Taiwan; 6 School of Post-Baccalaureate Chinese Medicine, Tzu Chi University, Hualien 97004, Taiwan

**Keywords:** hepatocellular carcinoma, sequential therapy, meta-analysis, sorafenib, lenvatinib

## Abstract

**Simple Summary:**

Although atezolizumab plus bevacizumab is the standard first-line treatment for patients with advanced HCC, the optimal second-line regimen remains unknown. In this meta-analysis, we examined the subsequent regimen after the failure of atezolizumab–bevacizumab and found that sequential multitargeted tyrosine kinase inhibitors, including sorafenib and lenvatinib, significantly prolonged the survival of patients, with a pooled OS of 11.45 months and a PFS of 3.78 months, respectively. Moreover, the subsequent use of sorafenib or lenvatinib has manageable toxicity. Therefore, our data indicate that sorafenib or lenvatinib treatment is a rational option for patients with advanced HCC after progression on atezolizumab–bevacizumab.

**Abstract:**

Background: Although atezolizumab plus bevacizumab (hereinafter, atezolizumab–bevacizumab) is the standard first-line treatment for patients with advanced HCC, the optimal second-line regimen remains unknown. This study evaluated the efficacy and safety of sorafenib and lenvatinib in patients with advanced HCC that progressed under atezolizumab–bevacizumab treatment. Methods: Following the Preferred Reporting Items for Systematic Reviews and Meta-Analyses guidelines, we searched PubMed, Embase, and the Cochrane Library for articles published before November 2023. Random-effects meta-analysis was performed to determine the pooled objective response rate (ORR), disease control rate (DCR), progression-free survival (PFS), and overall survival (OS), comparing patients who received sorafenib versus lenvatinib. Results: Seven studies involving 387 patients were included. The pooled ORR, DCR, OS, and PFS for sorafenib and lenvatinib together were 26% (95% CI: 14–43%), 63% (95% CI: 47–77%), 11.45 months (95% CI: 7.12–15.77, *I*^2^ = 92%, *p* < 0.01), and 3.78 months (95% CI: 2.34–5.23, *I*^2^ = 67%, *p* = 0.02), respectively. Although lenvatinib users had a longer median OS (12.42 vs. 10.75 months) and PFS (5.15 vs. 2.58 months) than sorafenib users, the pooled ORR, DCR, median OS, and PFS for these medications were comparable. Additionally, the distributions of all-grade and grade ≥ 3 adverse events for sorafenib and lenvatinib were comparable to those for these two medications when used as first-line therapies. Conclusions: Sorafenib or lenvatinib can provide effective treatment with manageable toxicity in patients with advanced HCC after disease progression under atezolizumab–bevacizumab.

## 1. Background

Hepatocellular carcinoma (HCC) is the sixth most common cancer and the fourth most common cause of cancer-related mortality worldwide [1]. The prognosis of patients with HCC is usually poor because most patients receive the diagnosis when the cancer is at an advanced stage, their underlying liver function has deteriorated, and their liver tumors have progressed; few patients with disease at this advanced stage are eligible for curative or liver-directed treatments such as surgical resection, radiofrequency ablation, liver transplantation, transarterial chemoembolization, and radioembolization.

Systemic therapy has become the cornerstone of treatment for patients with intermediate or advanced HCC whose condition does not respond to traditional treatments or who are unsuitable for transarterial chemoembolization [2]. Several systemic therapies are available. In particular, the combination of atezolizumab (which targets programmed death ligand 1 [PD-L1]) and bevacizumab (anti-vascular therapy) has, since 2020, been endorsed by several major international societies as a first-line treatment for patients with advanced HCC, including the American Association for the Study of Liver Disease (AASLD) [3], International Liver Cancer Association (ILCA), European Association for the Study of the Liver (EASL) [4], Asia Pacific Association for the Study of the Liver (APASL) [5], and European Society for Medical Oncology (ESMO) [6,7].

Although the combination of atezolizumab plus bevacizumab (hereinafter, atezolizumab–bevacizumab) can significantly prolong overall survival (OS) and progression-free survival (PFS) and improve the quality of life of patients with advanced HCC [7,8], its efficacy remains unsatisfactory; approximately 60% of patients experience disease progression or die after receiving this regimen [7]. Therefore, selection of the optimal regimen after first-line atezolizumab–bevacizumab has failed and the HCC has progressed is a critical concern in the management of advanced HCC. One option is to employ the former first-line treatment, multitargeted tyrosine kinase inhibitors (MKIs) such as sorafenib and lenvatinib [9,10], as a second-line option [11,12]. Several studies have examined the use of MKIs in patients with advanced HCC after disease progression with atezolizumab–bevacizumab and have obtained promising results [13,14]. However, the sample sizes in these studies were small enough that the results must be interpreted cautiously.

In this study, we performed a systematic review and meta-analysis to evaluate the efficacy and safety of MKIs in patients with advanced HCC that progressed after failure of treatment with atezolizumab–bevacizumab.

## 2. Materials and Methods

### 2.1. Data Sources and Search Strategy

This systematic review and meta-analysis followed the latest Preferred Reporting Items for Systematic Reviews and Meta-Analyses (PRISMA) guidelines [15]. The study was registered with PROSPERO (registration number: CRD42023488252). Two authors (Y.-F.W. and T.-R.P.) searched PubMed, the Cochrane Library, and Embase (OVID) for relevant articles published before 1 November 2023. The search strategy used was (“atezolizumab” OR “tecentriq”) AND (“bevacizumab” OR “avastin”) AND (“hepatocellular carcinoma” OR “HCC”) AND (“sorafenib” OR “lenvatinib”). Abstracts from annual meetings of the AASLD, ILCA, EASL, APASL, and ESMO were also searched for relevant data.

We included randomized controlled trials (RCTs) and prospective and observational studies (either cohort or case-control) that evaluated the effects of sorafenib or lenvatinib in patients with advanced HCC after the failure or progression of treatment with first-line atezolizumab–bevacizumab. Two reviewers (Y.-F.W. and T.-R.P.) independently screened all titles and abstracts and evaluated relevant articles. C.-S.H. served as the final reviewer. 

### 2.2. Inclusion and Exclusion Criteria

Studies were included if they (1) enrolled patients with a diagnosis of HCC; (2) enrolled patients receiving sorafenib or lenvatinib after the failure or progression of treatment with first-line atezolizumab–bevacizumab (as determined according to RECIST and its modification criteria); (3) documented the occurrence of any clinical tumor outcome mentioned in the literature, such as the overall response rate (ORR), disease control rate (DCR), median OS, median PFS, and adverse events (AEs); and (4) were RCTs or prospective or retrospective studies published in English, including single-arm studies. Studies were excluded if they (1) were an animal experiment, case report, review, letter, comment, or editorial; (2) were published in a language other than English; or (3) contained incomplete data. 

### 2.3. Data Extraction

Data were independently extracted, analyzed, and recorded on a predeveloped data extraction sheet by two reviewers (T.-W.W. and T.-R.P.). The final decision was made after consultation with a third reviewer (C.-S.H.), and a group consensus was then reached. We extracted the first author, published year, study design, treatment regimen, sample size, measured results (ORR, DCR, OS, and PFS), and details on AEs from the studies. The hazard ratios of the time-to-event variables (OS and PFS) were extracted directly from the original studies or estimated indirectly by using the number of events and the corresponding *p*-values for the log-rank statistics.

### 2.4. Quality Assessment of Included Studies

Two reviewers (T.-W.W. and T.-R.P.) separately assessed the quality of the included studies. For RCTs, the revised risk-of-bias 2.0 method (version 2.0) was used to categorize bias as low, unclear, or high (green, yellow, or red) in each study. We used the Newcastle–Ottawa scale to assess the quality of observational studies [16]. The total score on this scale is 9 points. High-quality results are rated 6 points or higher. The quality of the included retrospective single-arm studies was assessed using the JBI Critical Appraisal Checklist for Case Series [17].

### 2.5. Statistical Analyses

All statistical analyses were performed using Stata version 15.0 (Stata, College Station, TX, USA). Due to the observational, single-arm, noncomparative nature of the dataset, a standard meta-analysis was not statistically feasible. An alternative to standard meta-analysis is a meta-analysis of proportions, where the weighted proportion of a binary outcome variable is determined by the average proportions of studies weighted using inverse sampling variance. The meta-analysis of proportions in the present study was performed using the methods previously described [18,19]. When the data were observational or included two arms, we used the statistical techniques of a standard meta-analysis. We calculated the pooled odds ratio (OR) and the 95% CI for ORR, DCR, and AEs. Calculations were performed using a DerSimonian–Laird random-effects meta-analysis [20] under the assumption of significant heterogeneity. Heterogeneity among studies was quantified using the *I*^2^ test, and *I*^2^ > 50% was considered substantial heterogeneity. A *p*-value < 0.10 was considered statistically significant. Publication bias was analyzed using Egger’s test and Begg’s test. All statistical analyses were performed in accordance with the procedures in the Cochrane Handbook for the Statistical Review of Interventions (version 6.2) [15]. 

## 3. Results

### 3.1. Selection of Studies

**Figure 1:**
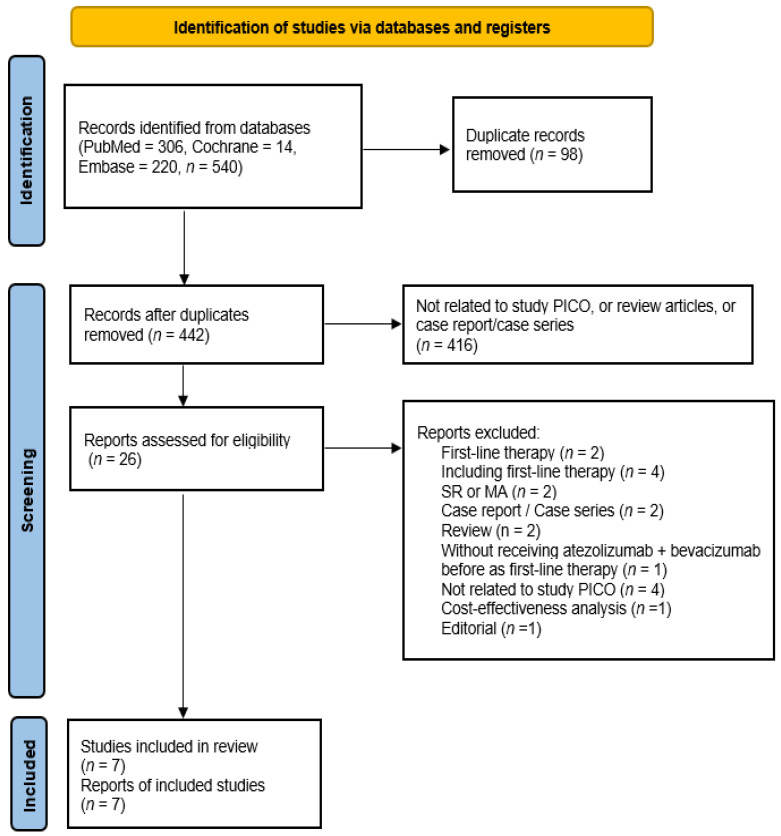
Preferred Reporting Items for Systematic Reviews and Meta-Analyses (PRISMA) flow diagram for study selection [15].

We identified 540 records from the PubMed, EMBASE, and Cochrane electronic databases. Ninety-eight studies were removed as duplicates. After excluding these studies, we had 442 remaining and reviewed them on the basis of their titles and abstracts; a further 416 studies were thereby excluded due to the irrelevance of their titles to the subject of our analysis. Finally, we subjected the remaining 26 studies to full-text screening and excluded 19 articles because of irrelevant content. Finally, seven studies met our inclusion criteria. The PRISMA flowchart presented in Figure 1 shows the detailed process of study selection.

### 3.2. Characteristics of Eligible Studies

Seven retrospective studies (including two multi-country and multi-center studies) conducted over a period from 2021 to 2023 and involving 555 patients receiving atezolizumab–bevacizumab treatment (including 387 patients received further treatment: lenvatinib [*n* = 255], sorafenib [*n* = 132], cabozantinib [*n* = 28], and ramucirumab [*n* = 29]) were included in this meta-analysis. The characteristics of the included studies are presented in Table 1. The results of the quality assessment of the seven single-arm retrospective studies are shown in Table 2. 

### 3.3. Response to Treatment Following Failure of Atezolizumab–Bevacizumab Therapy 

**Figure 2:**
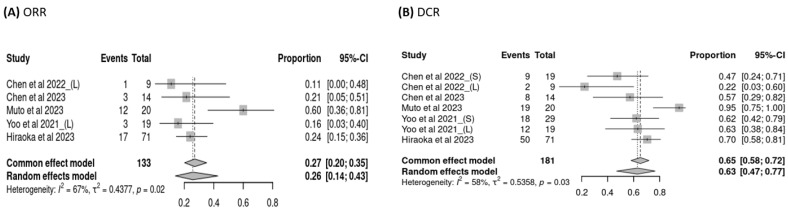
Pooled (**A**) objective response rate (ORR) and (**B**) disease control rate (DCR) of sorafenib and lenvatinib.

**Figure 3:**
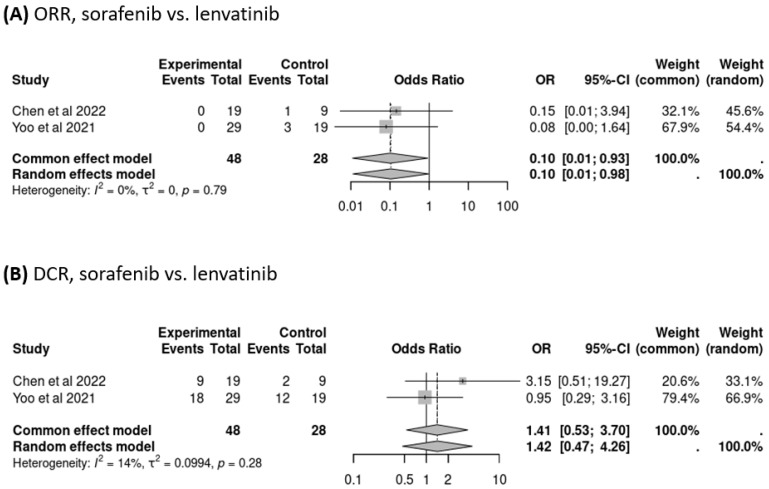
Comparison of objective response rate (ORR) and disease control rate (DCR) of treatment with sorafenib versus lenvatinib.

This study analyzed the pooled ORR and DCR of treatment with sorafenib or lenvatinib after the failure of atezolizumab–bevacizumab. ORR data from five trials (*n* = 133 patients) were available for analysis, and the pooled ORR was 26% (95% CI: 14–43%), as determined by a random-effects model (heterogeneity analysis: *I*^2^ = 67%, *p* = 0.02; Figure 2A). DCR data from seven trials (*n* = 181 patients) were available for analysis, and the pooled DCR was 63% (95% CI: 47–77%), as determined by a random-effects model (heterogeneity analysis: *I*^2^ = 58%, *p* = 0.03; Figure 2B). Because most patients received sorafenib or lenvatinib after the failure of atezolizumab–bevacizumab treatment (*n* = 555), we further examined the ORR and DCR of users of sorafenib versus lenvatinib, which were found to be comparable (OR: 0.10, 95% CI: 0.01–0.98, *p* = 0.79; OR: 1.42, 95% CI: 0.47–4.26, *p* = 0.28, respectively; Figure 3A,B).

### 3.4. Treatment Survival Outcomes Following the Failure of Atezolizumab–Bevacizumab Therapy

**Figure 4:**
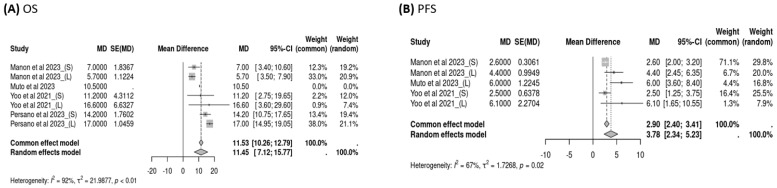
Pooled overall survival (OS) and progression-free survival (PFS) for treatment with sorafenib or lenvatinib.

**Figure 5:**
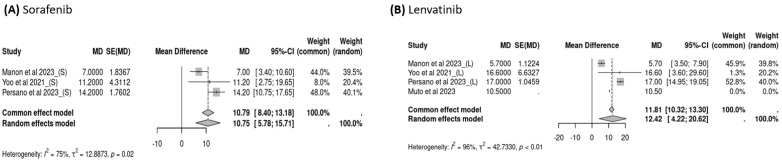
Overall survival (OS) for treatment with sorafenib versus lenvatinib.

**Figure 6:**
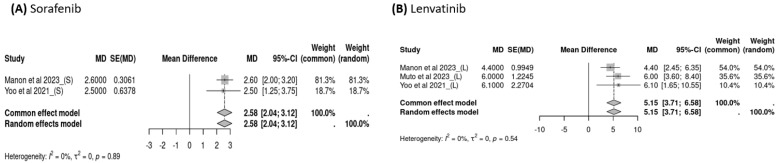
Progression-free survival (PFS) for treatment with sorafenib versus lenvatinib.

Three studies reported OS data for subsequent treatments after the failure of atezolizumab–bevacizumab, and the pooled OS of subsequent treatment was 11.45 months (95% CI: 7.12–15.77, *I*^2^ = 92%, *p* < 0.01; Figure 4A) overall, 10.75 months (95% CI: 5.78–15.71, *I*^2^ = 75%, *p* = 0.02; Figure 5A) for sorafenib users, and 12.42 months (95% CI: 4.22–20.62, *I*^2^ = 96%, *p* < 0.01; Figure 5B) for lenvatinib users. Three studies examined PFS data following the failure of atezolizumab–bevacizumab treatment, and the pooled PFS for subsequent treatment was 3.78 months (95% CI: 2.34–5.23, *I*^2^ = 67%, *p* = 0.02; Figure 4B) overall, 2.58 months (95% CI: 2.04–3.12, *I*^2^ = 0%, *p* = 0.89; Figure 6A) for sorafenib users, and 5.15 months (95% CI: 3.71–6.58, *I*^2^ = 0%, *p* = 0.54; Figure 6B) for lenvatinib users. Although the use of lenvatinib was associated with a longer OS and PFS than was the use of sorafenib, the difference was nonsignificant (*p* > 0.05). 

### 3.5. AEs

**Figure 7:**
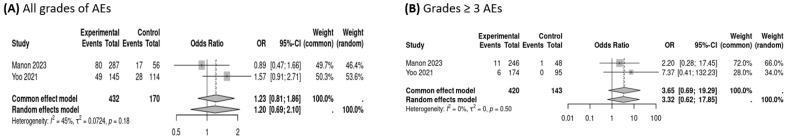
Pooled incidence of all-grade and grade ≥ 3 adverse events (AEs) for treatment with sorafenib versus lenvatinib.

Five studies reported the incidence of all-grade AEs and grade ≥ 3 AEs [13,21,22,23,25], including a couple of two-arm studies comparing the AEs of sorafenib and lenvatinib [13,22]. The most common all-grade AEs were hand–foot syndrome (43%), fatigue (41%), an elevated aspartate or alanine aminotransferase level (35%), hypertension (23%), diarrhea (22%), nausea (12%), and rash (10%; Table 3). However, the pooled incidences of all-grade AEs and grade ≥ 3 AEs were comparable for sorafenib and lenvatinib (OR: 1.20, 95% CI: 0.69–2.10, *p* = 0.18; OR: 3.32, 95% CI: 0.62–17.85, *p* = 0.50, respectively; Figure 7A,B), and the distributions of all-grade and grade ≥ 3 AEs for sorafenib and lenvatinib were comparable to those reported when these medications were used as first-line therapies [14,27]. 

### 3.6. Sensitivity Analysis

We performed a sensitivity analysis by excluding the studies with a wide 95% CI [13]; the results revealed a pooled OS and PFS of 11.03 months (95% CI: 5.52–16.54, *I*^2^ = 93.6%, *p* < 0.001) and 3.48 months (95% CI: 2.22–4.74, *I*^2^ = 70.0%, *p* = 0.0018) for sorafenib and lenvatinib, respectively.

### 3.7. Publication Bias

Egger’s and Begg’s tests were used to detect publication bias in the meta-analysis; no significant publication bias was discovered for the pooled ORR (Egger’s test and Begg’s test: *p* = 0.383 and 0.50, respectively) or pooled DCR (Egger’s test and Begg’s test: *p* = 0.354 and *p* = 0.382, respectively).

## 4. Discussion

Although atezolizumab–bevacizumab is the standard first-line therapy for patients with advanced HCC, the optimal subsequent treatment and salvage regimen for when this treatment fails has yet to be determined [7]. In this systematic review and meta-analysis, we examined the subsequent regimen after the failure of atezolizumab–bevacizumab and found that sequential MKIs, including sorafenib and lenvatinib, significantly prolonged the survival of patients, with a pooled OS of 11.45 months and a PFS of 3.78 months. Moreover, the subsequent use of sorafenib or lenvatinib caused manageable toxicity, similar to that when these medications were used as first-line therapies. Therefore, our data indicate that sorafenib or lenvatinib treatment is a rational option for patients with advanced HCC after progression on atezolizumab–bevacizumab.

Because approximately 70–80% of patients fail to respond to atezolizumab–bevacizumab treatment [7,8], identifying the optimal sequential strategy after disease progression on atezolizumab–bevacizumab is critical in real-world clinical practice. Although several regimens have been tested—including sorafenib, lenvatinib, regorafenib, cabozantinib, and ramucirumab [28]—no randomized controlled clinical trials comparing the efficacy of various sequential treatments have been conducted; most studies have adopted a retrospective design with small sample sizes and thus could not reach definite conclusions [28]. In this systematic review and meta-analysis, we examined the results of these studies; most employed are sorafenib and lenvatinib, with a pooled ORR and DCR for the salvage use of sorafenib or lenvatinib following HCC progression on atezolizumab–bevacizumab of 26% and 62%, respectively. The pooled mean OS was 11.45 months, and the pooled mean PFS was 3.78 months. These results are similar to the prospective and real-world data of studies using MKIs as first-line therapies [14,27], indicating that the efficacy of MKIs may not be affected by previous atezolizumab–bevacizumab treatment. Further research is required to clarify the underlying molecular pathological mechanisms for the salvage use of MKIs after atezolizumab–bevacizumab treatment.

In this study, although lenvatinib users had slightly superior median OS (12.42 months) and PFS (5.15 months) to sorafenib users, the difference between sorafenib and lenvatinib as second-line therapies after progression on atezolizumab–bevacizumab was nonsignificant (ORR and DCR: *p* = 0.79 and *p* = 0.28). However, these findings must be interpreted cautiously because our study is a retrospective analysis of real-world data; confounding factors may be present, and the baseline characteristics of sorafenib versus lenvatinib users may not have been well matched. Future RCTs with well-matched baseline patients are warranted to clarify the efficacy of second-line treatment with sorafenib and lenvatinib in patients with advanced HCC.

Although potential unexpected toxicities after the discontinuation of primary immunotherapy [29] were a concern in many of the reviewed studies, the safety profiles of sorafenib and lenvatinib after atezolizumab–bevacizumab treatment were comparable to those reported for these medications when used as first-line therapies [14,26]. The most common AEs were hand–foot syndrome (43%) and fatigue (41%), and grade ≥3 AEs occurred in fewer than 10% of enrolled patients. Moreover, no significant differences were found in all-grade and grade ≥ 3 AEs between users of sorafenib and lenvatinib [13,22]. Due to their therapeutic efficacy, manageable toxicity, and familiarity in clinical use, sorafenib and lenvatinib may become widely used regimens after the failure of atezolizumab–bevacizumab [27]. However, several other potential regimens are being developed or tested, such as other MKIs, PD-1/PD-L1 blockades, and anti-cytotoxic T lymphocyte-associated protein 4 antibody. Thus, future large-scale, cross-regional studies are warranted to identify the optimal regimen for patients with HCC after the failure of atezolizumab–bevacizumab.

This meta-analysis has some limitations. First, we were unable to locate any RCTs that examined specific second-line regimens after the failure of atezolizumab–bevacizumab; thus, this systematic review only included retrospective studies with clinical cohorts. Second, because the numbers of articles and patients included in each study were relatively small, the heterogeneity among the included studies was significant. Third, the included studies used slightly different methods to assess treatment response. Five of them used RECIST v 1.1. [13,21,22,23,26], and the other two used RECIST and its modification [24,25]. However, after we performed a sensitivity analysis to exclude these two studies, the results for ORR and DCR were still similar (ORR: 21%, 95% CI: 15–30%; DCR: 59%, 95% CI: 47–70%; respectively).

Finally, because few studies examined the sequential use of other MKIs (regorafenib or cabozantinib), ramucirumab, PD-1/PD-L1 blockade, or anti-cytotoxic T lymphocyte-associated protein 4 antibody after the failure of atezolizumab–bevacizumab, we could not investigate the clinical outcomes of the sequential use of other potential candidate treatments.

## 5. Conclusions

This systematic review and meta-analysis determined that, in patients with HCC for whom atezolizumab–bevacizumab does not prevent disease progression, treatment with sorafenib or lenvatinib has manageable toxicity and can significantly prolong survival. Therefore, sorafenib or lenvatinib treatment is a rational option as a second-line treatment after failure of atezolizumab–bevacizumab. Considering the various potential candidate regimens in development, future studies with more data are required to determine the optimal therapy sequence after the failure of atezolizumab–bevacizumab.

## Figures and Tables

**Table 1:** Characteristics of included studies.

Author/Year	Study Design	Period	Country	No. of Centers	No. of Patients	Intervention (Patients)	Comparator (Patients)	ORR/DCR	OS, Months (Median, 95% CI)	PFS, Month (Median, 95% CI)
Chen et al., 2022 [21]	Retrospective	January 2018–May 2021	Taiwan	4	41	Sorafenib (19), Lenvatinib (9), Others (2), Not received second-line therapy (11)	NA	ORR S: 0/19; L: 1/9 DCR S: 9/19; L: 2/9	S: 8.3 L: 3.8	S: 2.6 L: 2.0
Chen et al., 2023 [22]	Retrospective	January 2020–September 2022	Taiwan	1	27	Lenvatinib (14), Others (13)	NA	ORR L: 3/14 DCR L: 8/14	L: 3.3	L: 4.2
Manon et al., 2023 [23]	Retrospective	April 2020–June 2022	France	11	82	Regorafenib (29)	Sorafenib (41), Lenvatinib (8), Cabozantinib (4)	NA	S: 7.0 (4.4–11.6) L: 5.7 (4.7–9.1)	S: 2.6 (2.2–3.4) L: 4.4 (1.8–5.7)
Muto et al., 2023 [24]	Retrospective	February 2021–May 2023	Japan	1	20	Lenvatinib (20)	NA	ORR L: 12/20 DCR L: 19/20	L: 10.5 (6.9–NR)	L: 6.0 (3.0–7.8)
Yoo et al., 2021 [13]	Retrospective	July 2016–April 2019	Korea, Hong Kong, Singapore	3	49	Sorafenib (29), Lenvatinib (19), Cabozantinib (1)	NA	ORR S: 0/29; L: 3/19 DCR S: 18/29; L: 12/19	S: 11.2 (2.7–19.6) L: 16.6 (3.6–29.6)	S: 2.5 (1.3–3.8) L: 6.1 (1.6–10.5)
Persano et al., 2023 [25]	Retrospective	July 2010–May 2022	Multi-Country	Multi-Center	206	NA	Sorafenib (43), Lenvatinib (84), Cabozantinib (23), Others (56)	NA	S: 14.2 (8.8–15.7) L: 17.0 (14.8–18.9)	NA
Hiraoka et al., 2023 [26]	Retrospective	2020–2022	Japan	1	130	Lenvatinib (101)	Others (29)	ORR L: 17/71 DCR L: 50/71	L: 13.6	L: 3.5

Abbreviations: NA: not applicable. ORR: overall response rate. DCR: disease control rate. OS: overall survival. PFS: progression-free survival.

**Table 2:** Quality assessment of included studies.

The JBI Critical Appraisal Checklist for Case Series’ Included Retrospective Single-Arm Studies											
Study	Q1	Q2	Q3	Q4	Q5	Q6	Q7	Q8	Q9	Q10	Overall Appraisal
Chen et al., 2022 [20]	Yes	Yes	Yes	Yes	Yes	Yes	Yes	Yes	Yes	Yes	Include
Chen et al., 2023 [21]	Yes	Yes	Yes	Yes	Yes	Yes	Yes	Yes	Yes	Yes	Include
Manon et al., 2023 [22]	Yes	Yes	Yes	Yes	Yes	Yes	Yes	Yes	Yes	Yes	Include
Muto et al., 2023 [23]	Yes	Yes	Yes	Yes	Yes	Yes	Yes	Yes	Yes	Yes	Include
Yoo et al., 2021 [13]	Yes	Yes	Yes	Yes	Yes	Yes	Yes	Yes	Yes	Yes	Include
Persano et al., 2023 [24]	Yes	Yes	Yes	Yes	Yes	Yes	Yes	Yes	Yes	Yes	Include
Hiraoka et al., 2023 [25]	Yes	Yes	Yes	Yes	Yes	Yes	Yes	Yes	Yes	Yes	Include

Q1. Were there clear criteria for inclusion in the case series? Q2. Was the condition measured in a standard, reliable way for all participants included in the case series? Q3. Were valid methods used for the identification of the condition for all participants included in the case series? Q4. Did the case series have consecutive inclusion of participants? Q5. Did the case series have complete inclusion of participants? Q6. Was there clear reporting of the demographics of the participants in the study? Q7. Was there clear reporting of clinical information on the participants? Q8. Were the outcomes or follow-up results of cases reported? Q9. Was there clear reporting of the presenting site(s)/clinic(s) demographic information? Q10. Was the statistical analysis appropriate?

**Table 3:** Pooled results of common adverse events.

Adverse Event	All Grade		≥Grade 3	
	ES, % (95% CI)	I2, %	ES, % (95% CI)	I2, %
Hand–foot syndrome	43 (27–59)	72.7	8 (2–15)	0
Fatigue	41 (23–59)	87.7	6 (2–9)	0
Elevated aspartate or alanine aminotransferase	35 (1–69)	96.1	N	N
Hypertension	23 (13–34)	56.8	4 (0–8)	0
Diarrhea	22 (15–30)	95.5	5 (−1–1)	0
Nausea	12 (2–22)	19.4	2 (−2–7)	100
Rash	10 (4–15)	0	5 (−2–11)	100

Abbreviations: ES: effect size. CI: confidence interval.
